# BK_Ca_ Activator NS1619 Improves the Structure and Function of Skeletal Muscle Mitochondria in Duchenne Dystrophy

**DOI:** 10.3390/pharmaceutics14112336

**Published:** 2022-10-29

**Authors:** Mikhail V. Dubinin, Vlada S. Starinets, Natalia V. Belosludtseva, Irina B. Mikheeva, Yuliya A. Chelyadnikova, Anastasia D. Igoshkina, Aliya B. Vafina, Alexander A. Vedernikov, Konstantin N. Belosludtsev

**Affiliations:** 1 Department of Biochemistry, Cell Biology and Microbiology, Mari State University, pl. Lenina 1, 424001 Yoshkar-Ola, Russia; 2 Laboratory of Mitochondrial Transport, Institute of Theoretical and Experimental Biophysics, Russian Academy of Sciences, Institutskaya 3, 142290 Pushchino, Russia

**Keywords:** Duchenne muscular dystrophy, *mdx*, skeletal muscle mitochondria, potassium transport, BK_Ca_, NS1619, MPT pore

## Abstract

Duchenne muscular dystrophy (DMD) is a progressive hereditary disease caused by the absence of the dystrophin protein. This is secondarily accompanied by a dysregulation of ion homeostasis, in which mitochondria play an important role. In the present work, we show that mitochondrial dysfunction in the skeletal muscles of dystrophin-deficient *mdx* mice is accompanied by a reduction in K^+^ transport and a decrease in its content in the matrix. This is associated with a decrease in the expression of the mitochondrial large-conductance calcium-activated potassium channel (mitoBK_Ca_) in the muscles of *mdx* mice, which play an important role in cytoprotection. We observed that the BK_Ca_ activator NS1619 caused a normalization of mitoBK_Ca_ expression and potassium homeostasis in the muscle mitochondria of these animals, which was accompanied by an increase in the calcium retention capacity, mitigation of oxidative stress, and improvement in mitochondrial ultrastructure. This effect of NS1619 contributed to the reduction of degeneration/regeneration cycles and fibrosis in the skeletal muscles of *mdx* mice as well as a normalization of sarcomere size, but had no effect on the leakage of muscle enzymes and muscle strength loss. In the case of wild-type mice, we noted the negative effect of NS1619 manifested in the inhibition of the functional activity of mitochondria and disruption of their structure, which, however, did not significantly affect the state of the skeletal muscles of the animals. This article discusses the role of mitoBK_Ca_ in the development of DMD and the prospects of the approach associated with the correction of its function in treatments of this secondary channelopathy.

## 1. Introduction

Duchenne muscular dystrophy (DMD) is a recessive X-linked hereditary disease caused by mutations (most often frameshifting deletions and insertions) in the DMD gene encoding the dystrophin protein. This is one of the most common forms of muscular dystrophy; on average, DMD is diagnosed in 1 in 3500 boys [1]. The dystrophin protein is part of the costamer, a protein complex responsible for the relationship of sarcomeres, the plasma membrane of the muscle fiber, and extracellular matrix proteins; it connects the neuronal nitric oxide synthetases (nNOS) with the sarcolemma through the glycoprotein complex, regulating blood flow to the muscles [2]. Furthermore, members of the glycoprotein complex are major components of various signaling pathways [3,4]. In the absence of dystrophin, muscle fibers become brittle, which causes rupture of the sarcolemma, an increase in their permeability during muscle contraction, the release of soluble enzymes such as creatine kinase from cells, and the penetration of calcium and other ions into cell [5]. Immune cell invasion and muscle injury lead to massive inflammation, apoptosis, and necrosis [6]. Cells show developments of oxidative stress, which also contribute to muscle damage [7]. Lack of dystrophin is also a cause of cardiomyopathy [8]. The average life expectancy of patients with DMD is approximately 19 years without treatment, but the use of corticosteroids, mechanical ventilation during sleep, and exercise therapy can extend it to 30 years [9].

Currently, there are ongoing clinical trials aimed at creating a gene therapy that can restore the normal expression of dystrophin [10,11]. However, such approaches often face multiple technical problems, primarily due to vector delivery, and can only be effective if therapy is started early, i.e., before any irreversible replacement of muscle tissue with fibrous deposits has occurred. In this regard, much attention is still paid to the correction of the secondary effects of DMD, primarily the disturbance of calcium homeostasis associated with an increase in reactive oxygen species (ROS), inflammation, regenerative malfunction, and fibrosis [12]. On the one hand, these phenomena are considered to be initiated by an abnormal increase of store-operated Ca^2+^ entry through the plasma membrane cationic channels of muscle fibers [13,14]; on the other hand, they are initiated by depletion of Ca^2+^ from intracellular deposits, primarily in the sarcoplasmic reticulum and mitochondria [15,16,17]. It is possible to discuss the degree of involvement of these pathways in the dysregulation of calcium homeostasis; however, this results in increased intracellular Ca^2+^, which would then activate calpain, underlying the degeneration of myofibers in muscular dystrophy.

Mitochondria, which provide muscle cells with the ATP necessary for normal contraction, deserve special attention. In the case of DMD, these organelles show a significant decrease in OXPHOS intensity, overgeneration of ROS, and rearrangements of calcium-transporting systems, including a dramatic reduction in calcium uniport efficiency and sensitivity to the induction of the mitochondrial permeability transition (MPT) pore, a non-selective protein channel in the inner and outer mitochondrial membranes [17,18,19,20]. In addition, DMD shows a reduction in the biogenesis of organelles and a violation of their dynamics [21,22,23]. Improving the functioning of the mitochondrial respiratory chain, the calcium-buffering capacity of mitochondria, and the activation of organelle biogenesis has been shown to lead to the normalization of mitochondrial function and decrease the intensity of destructive processes in skeletal muscles [23,24,25].

One of the possible therapeutic targets for the correction of mitochondrial dysfunctions is potassium ion homeostasis. The dysregulation of mitochondrial potassium channels plays an important role in the development of myopathies and, in particular, the dilated cardiomyopathy of various etiologies [26,27,28], which is also characteristic of Duchenne muscular dystrophy. Moreover, we have recently shown a reduction in mitochondrial potassium transport in the skeletal muscles of dystrophin-deficient *mdx* mice, which can be partially restored by a uridine treatment [29]. It is known that the inner membrane of mitochondria contains a number of potassium carriers [30]. Given the significant dysregulation of calcium-dependent processes, in this work we focused on the calcium-activated potassium channel (BK_Ca_), whose modulation (including that of the mitochondrial isoform (mitoBK_Ca_), protects cells from ROS overproduction and general mitochondrial dysfunction [31]. Therefore, we studied the effect of an intraperitoneal administration of benzimidazole derivative BK_Ca_ activator NS1619 (0.5 mg/kg per day) on the morphological and functional changes in the mitochondria, including the OXPHOS system, potassium transport and capacity, MPT pore opening, dynamics and biogenesis of organelles, and mitophagy; we also examined the overall state of the skeletal muscles of the dystrophin-deficient *mdx* mice. The results obtained indicate that NS1619 improves the functional activity of organelles and their ultrastructure, reduces the intensity of oxidative stress, and alleviates the destruction of skeletal muscles in *mdx* mice.

## 2. Materials and Methods

### 2.1. Animals

C57BL/10 (wild-type, WT) and dystrophin-deficient *mdx* (C57BL/10ScSn-*mdx*) mice were used in this study and were obtained from the Animal Breeding Facility, Branch of the Shemyakin and Ovchinnikov Institute of Bioorganic Chemistry, Russian Academy of Sciences, Russia (IBCh RAS Unique Research Device “Bio-model”). All the animals were singly housed and given a minimum of 72 h to acclimatize before manipulations. Mice had access to standard chow and water ad libitum. All of the animals were divided into four groups (*n* = 10 per each group): (1) wild-type mice treated with vehicle (WT); (2) WT + NS1619 (WT + NS); (3) *mdx* mice treated with vehicle; and (4) dystrophin-deficient mice treated with NS1619 (*mdx* + NS). Both strains of mice were treated starting at 8 weeks of age. 1,3-Dihydro-1-[2-hydroxy-5-(trifluoromethyl)phenyl]-5-(trifluoromethyl)-2H-benzimidazol-2-one or NS1619 (5 mg/mL, Sigma-Aldrich, St. Louis, MO, USA) was dissolved in a mixture of ethanol and sterile saline (7.4:92.6 *v*/*v*%) and interperitoneally administered in doses of 150–200 μL (0.5 mg/kg body weight) per animal every day for up to 4 weeks; control mice received solvent alone. All animals were sacrificed at the end of the experiment. The whole blood of each animal was collected for creatine kinase (CK), lactate dehydrogenase (LDH), and aspartate aminotransferase (AST) analyses using the appropriate kits (Vector-Best, Novosibirsk, Russia).

### 2.2. Mitochondria Isolation and Determination of Respiration and Oxidative Phosphorylation

Skeletal muscle mitochondria were isolated from the quadriceps of both hindlimbs as described earlier [17,18]. The isolation buffer contained the following reagents: 67 mM of sucrose, 10 mM of EDTA, 50 mM of KCl, 0.2% BSA, and 50 mM of Tris/HCl (pH 7.4). The resulting mitochondrial sample was resuspended in 250 mM of sucrose and 10 mM of Tris/HCl (pH 7.4) and contained 20–30 mg of mitochondrial protein/mL, as determined by the Bradford assay. The rate of oxygen consumption was polarographically recorded using the Oxygraph-2k respirometer (Oroboros Instruments, Innsbruck, Austria). The reaction buffer contained the following reagents: 120 mM of KCl, 5 mM of NaH_2_PO_4_, 2.5 mM of potassium glutamate, 2.5 of mM potassium malate, and 10 mM of Hepes/KOH (pH 7.4). We used 0.25 mg of mitochondrial protein/mL, 200 μM of ADP, and 50 μM of 2,4-dinitrophenol (DNP) in each experiment. The rates of oxygen consumption were calculated as nanomoles of O_2_/minute per 1 mg of protein, and the energetic state was determined as shown earlier [32,33].

### 2.3. Determination of Mitochondrial K^+^ Transport and Total Content

The rate of K^+^ transport across the inner mitochondrial membrane was estimated from the DNP-induced change in external K^+^ per minute (μM K^+^ ∗ min^−1^ ∗ mg^−1^ mitochondrial protein). The experimental buffer contained following reagents: 70 mM of mannitol, 180 mM of sucrose, 5 mM of NaH_2_PO_4_, 1 μg/mL of oligomycin, and 10 mM of Tris/HCl (pH 7.4). We used 0.25 mg of mitochondrial protein/mL and 50 μM of DNP in each experiment. The total potassium level was assessed by adding 0.1% triton X-100 detergent. 

### 2.4. Determination of Mitochondrial Ca^2+^ Retention Capacity, MPT Pore Opening Assay

The mitochondrial Ca^2+^ transport was spectrophotometrically recorded using an arsenazo III dye (675–685 nm) and Tecan Spark 10M plate reader (Tecan Group Ltd., Männedorf, Switzerland) [34]. To estimate the mitochondrial calcium retention capacity, 5 μM CaCl_2_ pulses were added into the reaction buffer containing 0.25 mg of mitochondrial protein/mL resuspended in 210 mM of mannitol, 70 mM of sucrose, 1 mM of KH_2_PO_4_, 2.5 of mM potassium glutamate, 2.5 mM of potassium malate, 10 μM of EGTA, 50 μM of arsenazo III and 10 mM of HEPES-KOH (pH 7.4). A spontaneous increase in external [Ca^2+^] after several Ca^2+^ pulses indicated MPT pore induction. The [Ca^2+^] released upon MPT pore opening was used as a measure of the calcium retention capacity (per milligrams of mitochondrial protein).

### 2.5. Assessment of Lipid Peroxidation

To assess the lipid peroxidation in skeletal muscle mitochondria, we spectrophotometrically measured the level of substances that react with thiobarbituric acid (TBARS). This assay measures the levels of malondialdehyde and other minor aldehyde forms that are interacting with thiobarbituric acid [35].

### 2.6. RNA Isolation, Reverse Transcription and Quantitative Real-Time PCR

100 mg of deep frozen quadriceps samples were taken for total RNA isolation using the ExtractRNA kit (#BC032, Eurogen, Moscow, Russia). A QuantStudio 1 amplifier (Thermo Fisher Scientific, Waltham, MA, USA) and a qPCRmix-HS SYBR reaction kit (Eurogen, Moscow, Russia) were used for the real-time PCR analysis. The Primer-BLAST tool was used to select and analyze gene-specific primers [36]. The oligonucleotide sequences of the PCR primers are shown in Table 1. The expression of each gene was normalized to the *Rplp2* mRNA level, and the comparative C_T_ approach was used to quantify the obtained data [37]. 

### 2.7. Quantification of Mitochondrial DNA

10 mg of quadriceps tissue (*vastus lateralis*) was taken for total DNA isolation (nuclear and mtDNA) using the DNA-Extran-2 kit (Sintol, Moscow, Russia). The total DNA was used for analysis in the amount of 1 ng. The PCR method was used to assess the content of mtDNA in the sample, as previously described [38]; the results were presented as the ratio of mtDNA to nuclear DNA. The ND4 gene of the mouse mitochondrial genome and the GAPDH gene encoded by the nuclear genome were selected for analysis. A comparison of ND4 DNA expression relative to GAPDH DNA expression allows for the estimation of the ratio of mtDNA copy number to nDNA copy number. The oligonucleotide sequences of the PCR primers for mtDNA and nDNA are shown in Table 1. A QuantStudio 1 amplifier (Thermo Fisher Scientific, Waltham, MA, USA) and a qPCRmix-HS SYBR reaction kit containing SYBR Green II DNA binding dye (Eurogen, Moscow, Russia) were used for the real-time PCR analysis.

### 2.8. Electron Microscopy

Pieces of the quadriceps muscle (two samples of *vastus lateralis* per group) were fixed in 0.1 M of PBS (pH 7.4) solution with the addition of 2.5% glutaraldehyde for 2 h. The sample was then fixed in PBS solution supplemented with 1% osmic acid and dehydrated by increasing the alcohol concentration. At the next stage, the samples were encapsulated in Epon 812 resin. Ultrathin sections that were 70–75 nm thick were made using a Leica EM UC6 microtome (Leica Microsystems, Wetzlar, Germany). The sections were stained with uranyl acetate and lead citrate. The skeletal muscle samples were visualized using a JEM-100B electron microscope (JEOL, Tokyo, Japan). An Epson V700 scanner was used to digitize the negatives, and the resulting images were analyzed using Image Tool 3.0 software.

### 2.9. Histological Examination of Skeletal Muscle Tissues

Samples of the skeletal muscle tissues (quadriceps, five samples per group) were used for the histological examination. All samples were preliminary fixed in a neutral buffered 10% formalin and were impregnated in paraffin wax. For the histological examination, sections were made with a thickness of 5 μm using the MC-2 microtome. Tissue staining was carried out according to the standard method of hematoxylin and eosin (H&E) and hematoxylin van Gieson (HvG) protocol. An EVOS M5000 imaging system (Thermo Fisher Scientific, Waltham, MA, USA) and the free ImageJ software were used to visualize and analyze the slides. The level of fibrosis in the muscles of mice was evaluated as the percentage ratio of the HvG staining areas (pink) representing collagen and other connective tissue elements in the common area of the tissue on the histological slides, wherein at least 10 sections were analyzed for each organ sample. Central nucleus fibers (CNF) stained with H&E were counted on average in 8–10 unique visual fields (20× objective) from each animal and were expressed as a percentage of the total number of myofibers counted. 

### 2.10. Wire-Hanging Test

The wire-hanging test [39] was used to evaluate the muscle function and endurance of the animals. To perform this, each animal was placed on a wire with a diameter of 3 mm and length of 38 cm, located at a height of 49 cm above a soft surface to absorb the fall. The task of the animal was to stay on the wire for 30 s. The test results were calculated as previously described [29].

### 2.11. Statistical Analysis

The data are expressed as the mean ± standard error of the mean (m ± SEM). A one-way ANOVA with Tukey’s post hoc test was performed using GraphPad Prism version 8.0.1 for Windows (GraphPad Software Inc., La Jolla, CA, USA). The differences between groups were declared statistically significant at *p* < 0.05.

## 3. Results

### 3.1. NS1619 Suppresses Respiration and OXPHOS in Skeletal Muscle Mitochondria of Dystrophin-Deficient and WT Mice

DMD is known to be related with dramatic dysfunction of skeletal muscle mitochondria due to the decreased intensity of oxidative phosphorylation [17,18,19,20]. As previously shown [17,18,29] and confirmed in the present work (Table 2), *mdx* mouse muscle mitochondria were characterized by a decrease in ADP-stimulated respiration (State 3) and a decrease in the maximum respiration rate in the presence of the uncoupler DNP (State 3U_DNP_). NS1619 caused a decrease in the state 4 respiration rate of *mdx* mouse mitochondria, and there was also a trend towards a further decrease in the rate of state 3 and 3U_DNP_ respiration. Moreover, in the case of wild-type animals, NS1619 caused a significant reduction in the rate of states 3 and 3U_DNP_ respiration. The use of NS1619 was accompanied by a significant decrease in the rate of state 2 mitochondrial respiration and the respiratory control ratio. Thus, NS1619 suppressed respiration and oxidative phosphorylation in skeletal muscle mitochondria, which was most pronounced in the wild-type animals, demonstrating higher respiratory rates compared with dystrophin-deficient mice. 

### 3.2. NS1619 Normalizes the Rate of Potassium Ion Transport and Total Ion Level in Skeletal Muscle Mitochondria of mdx Mice

**Figure 1:**
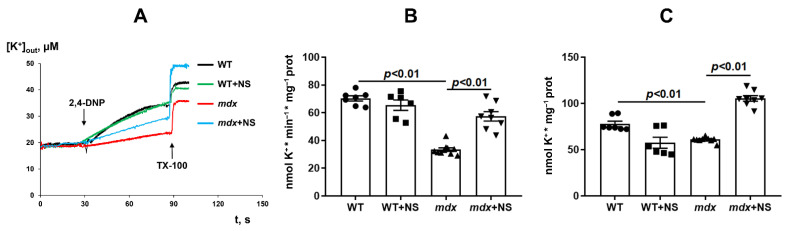
The effect of NS1619 (NS) on DNP-induced potassium efflux from mouse skeletal muscle mitochondria (**A**). Potassium release rate (**B**) and total content of potassium ions (**C**) in skeletal muscle mitochondria. Additions: 50 µM of 2,4-DNP and triton X-100 (TX-100, 0.1%). The data are presented as m ± SEM.

Because NS1619 is able to modulate potassium transport in mitochondria, the next part of the work evaluated the transport of this ion in the skeletal muscle mitochondria of the studied groups of animals. The transport of K^+^ ions was determined using an ion-selective K^+^ electrode by DNP-induced K^+^ release from mitochondria [40]. One can see that DMD animals demonstrated a two-fold decrease in the rate of transport of K^+^ ions from skeletal muscle mitochondria (Figure 1A,B). Moreover, we observed a decrease in total K^+^ in the skeletal muscle mitochondria of *mdx* mice (Figure 1A,C). Administration of NS1619 resulted in a significant increase in both the rate of DNP-induced release of K^+^ ions from the mitochondria of *mdx* mice and the total amount of mitochondrial K^+^.

**Figure 2:**
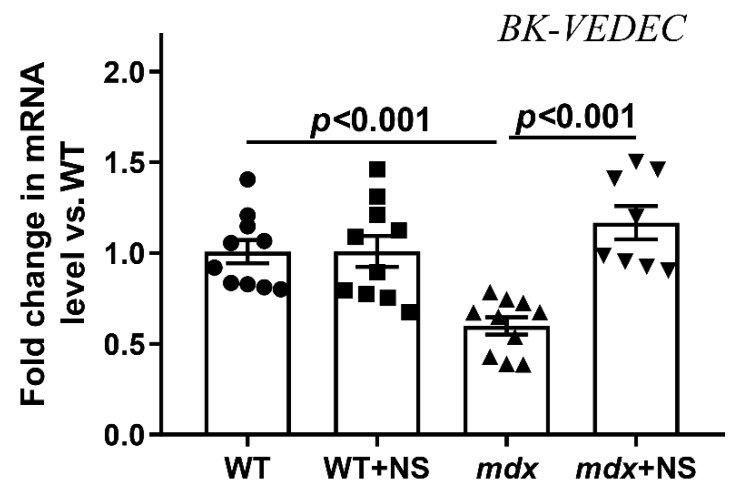
Relative level of BK-VEDEC mRNA (encoding mitoBK_Ca_ channel) in the skeletal muscle of experimental animals. The data are presented as m ± SEM.

We also assessed the expression level of the BK_Ca_ gene in the skeletal muscles of the experimental groups of mice. The current findings indicate rodent mitoBK_Ca_ is a splice variant of plasma membrane BK_Ca_ (from *Kcnma1* gene), which contains an extra 50 amino acids at the end of the C-terminus (BK-VEDEC sequence) that are essential to targeting BK_Ca_ to the mitochondria [41,42]. We have found that the expression of BK-VEDEC in the skeletal muscles of *mdx* mice was reduced by almost 1.7 times (Figure 2). In this case, treatment with NS1619 led to the normalization of BK-VEDEC expression in dystrophin-deficient animals.

### 3.3. NS1619 Increases Mitochondrial Calcium Retention Capacity and Attenuates Oxidative Stress in Skeletal Muscle Mitochondria of mdx Mice

**Figure 3:**
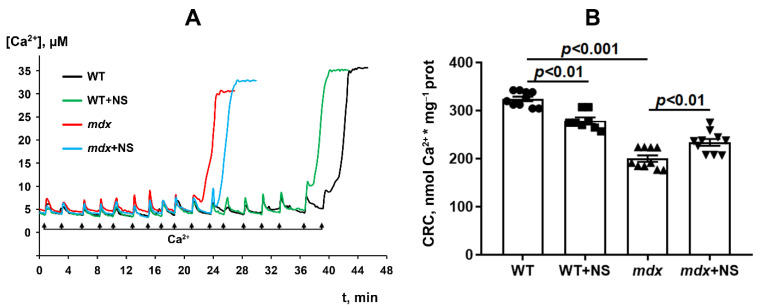
Changes in the external [Ca^2+^] upon the successive addition of 5 μM Ca^2+^ pulses to the suspension of the skeletal muscle mitochondria of the experimental animals (**A**). Ca^2+^ retention capacity of skeletal muscle mitochondria (**B**). The data are presented as m ± SEM.

It is well-known that dystrophin-deficient mice show a decrease in the resistance of skeletal muscle mitochondria to the induction of a calcium-dependent MPT pore, which can be recorded by a decrease in mitochondrial Ca^2+^ retention capacity. This was shown earlier [17,18,23,29] and was confirmed in the present work (Figure 3). It is believed that mitoBK_Ca_ channel regulation is closely related to MPT pore opening [31]. Indeed, pharmacological activation of the channel with NS1619 has been previously shown to increase the number of Ca^2+^ pulses necessary to cause a massive release of Ca^2+^ from the mitochondria [41], equal to MPT pore opening. In our case, we also noted an increase (approximately 1.2-fold) in the calcium retention capacity of the skeletal muscle mitochondria of *mdx* mice treated with NS1619 (Figure 3). At the same time, it should be noted that NS1619 on the contrary reduced the calcium retention capacity of mitochondria isolated from the skeletal muscles of wild-type animals.

**Figure 4:**
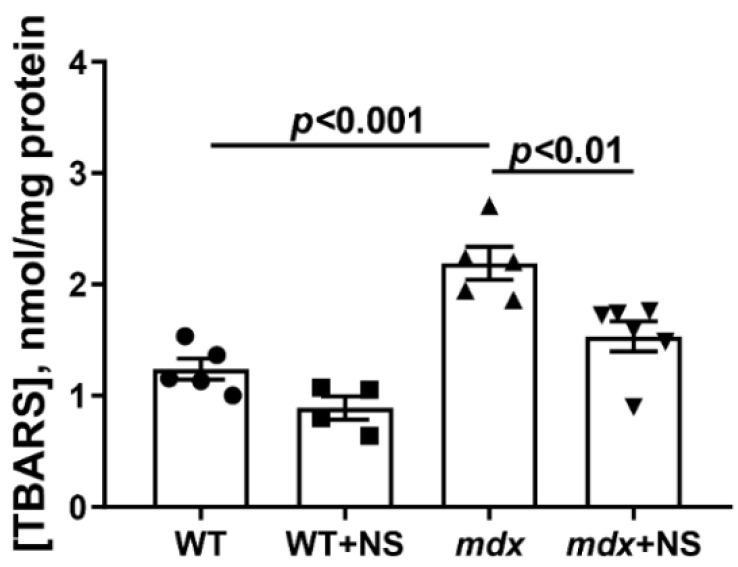
The effect of NS1619 on lipid peroxidation in mitochondria. Lipid peroxidation was assessed by the level of TBARS in the skeletal muscle mitochondria of the experimental animals. The data are presented as m ± SEM.

MPT pore induction in DMD is thought to be due to a complex interaction between excess Ca^2+^ and ROS [43,44]. The level of the latter, as well as their products, significantly increases with the development of this pathology [20,23,29]. Indeed, one can see that the skeletal muscle mitochondria of dystrophin-deficient animals show a 1.8-fold increase in TBARS compared with WT mice, indicating an intensification of lipid peroxidation in the mitochondrial membranes (Figure 4). Skeletal muscle mitochondria from *mdx* mice treated with NS1619 show a 1.4-fold reduction in TBARS levels. This confirms the well-known role of mitoBK_Ca_ in the regulation of ROS production [31].

### 3.4. NS1619 Normalizes the Ultrastructure of Skeletal Muscle Mitochondria and Sarcomere Size in mdx Mice but Does Not Affect the Expression of Protein Genes Responsible for Mitochondrial Biogenesis, Dynamics, and Mitophagy

**Figure 5:**
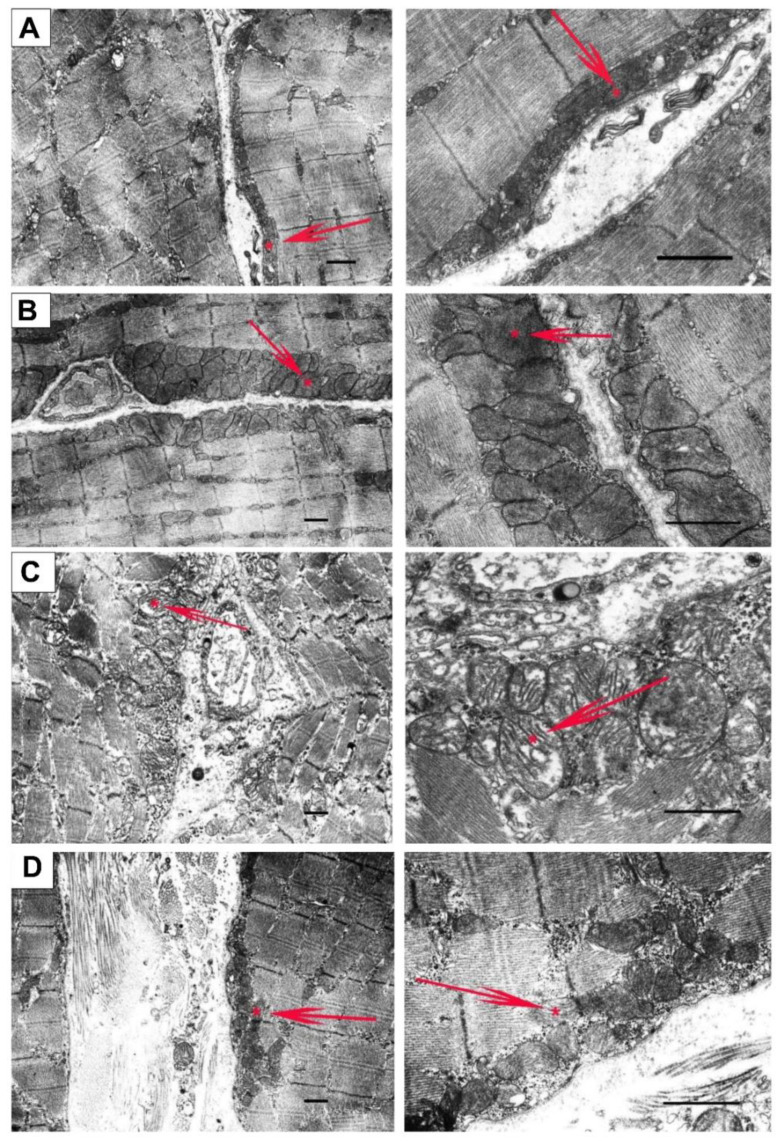
Representative electron micrographs of skeletal muscle tissue (quadriceps) from the WT (**A**), WT + NS1619 (**B**), *mdx* (**C**), and *mdx* + NS1619 (**D**) groups of mice. The red arrows highlight the subsarcolemmal population of mitochondria. An asterisk marks the same mitochondria at low and high magnification. The bar is equal to 1 μm.

**Figure 6:**
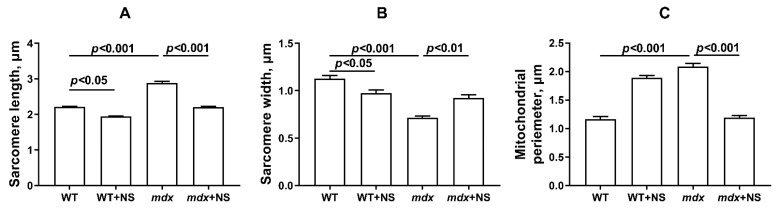
Graphical representation of electron micrograph (Figure 5) profiles: sarcomere length (**A**), sarcomere width (**B**), and mitochondrial perimeter (**C**). The number of examined fields of view varied from 40 to 50. The data are presented as m ± SEM.

Mitochondrial dysfunction in the skeletal muscle of *mdx* mice is also accompanied by changes in their ultrastructure. Figure 5 and Figure 6 show the electron microscopy data and morphometric parameters of the sarcomeres and subsarcolemmal population of mouse skeletal muscle mitochondria (Figure 5, red arrows).

In the control preparations (WT group), the skeletal muscles were characterized by a high-ordered packing of myofibrils, and mitochondria show a low distribution density under the sarcolemma. They have a dense matrix with well-packed cristae (Figure 5A). The WT + NS group (Figure 5B) also demonstrated a well-ordered packing of myofibrils, but we noted a statistically significant decrease in the size of the sarcomeres and the presence of areas of subsarcolemmal mitochondrial proliferation. Unusually large mitochondria appear (Figure 6), but matrix density and cristae packing do not differ from the WT group. The *mdx* group showed the heterogeneity of the intracellular organization, which was expressed in a decrease in the ordering of the myofibril packing and a sharp increase in the number of mitochondria with signs of pronounced destruction (Figure 5C). The sarcomeres show marked elongation and reduction in width (Figure 6A,B). It is possible to observe the hypertrophy of mitochondria due to their swelling (Figure 6C), violation of the structure of cristae, and matrix density becoming more enlightened and vacuolated. Accumulations of glycogen and lysosomes are often observed around pathologically altered mitochondria. At the same time, the *mdx* + NS group had a more ordered myofibril packing and a structure of the subsarcolemmal zone approaching the WT group (Figure 5D). Sections show a normalization of sarcomere size (Figure 6A,B). Mitochondria under the sarcolemma are not numerous and are characterized by an unchanged structure (Figure 6C). These data confirm the important role of mitoBK_Ca_ in maintaining mitochondrial structure and indicate that channel activation and restoration of its expression contribute to the normalization of the organelle ultrastructure and muscle sarcomere size.

**Figure 7:**
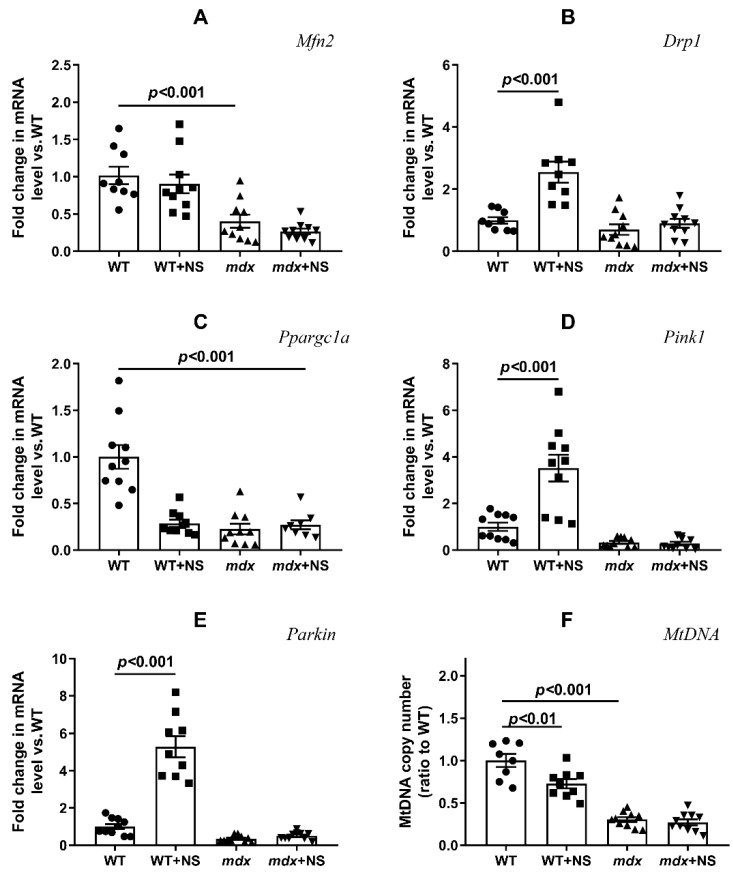
Effect of NS1619 on mitochondrial dynamics, biogenesis, and mitophagy in skeletal muscle. The relative mRNA levels of *Mfn2* (**A**), *Drp1* (**B**), *Ppargc1a* (**C**), *Pink1* (**D**), *Parkin* (**E**), and mtDNA (**F**) in the skeletal muscle of the experimental animals. The data are presented as m ± SEM.

We also assessed the expression levels of genes encoding proteins responsible for mitochondrial fusion (mitofusin 2) and fission (Drp1), organelle biogenesis (PGC1a), and mitophagy (Pink1 and Parkin). Figure 7 shows that the development of the *mdx* phenotype was accompanied by a decrease in the expression of the *Mfn2* and a decrease in the expression of the *Ppargc1a*. The expression of other genes in *mdx* mice did not change. NS1619 did not affect the level of expression of genes responsible for mitochondrial dynamics in the skeletal muscles of *mdx* mice, but it significantly increased the expression of the *Drp1* gene in the muscles of wild-type mice, which may indirectly indicate the activation of mitochondrial fission. NS1619 also had no effect on the expression of the *Ppargc1a* gene in *mdx* mice, which is responsible for organelle biogenesis; at the same time, NS1619 significantly reduced the expression level of this gene in the skeletal muscles of wild-type mice. When assessing the level of expression of the genes responsible for mitophagy, we noted that NS1619 did not affect the level of expression of these genes in the muscles of *mdx* mice but significantly increased the expression of the *Pink1* and *Parkin* genes in the skeletal muscles of wild-type mice, which may indirectly indicate the activation of mitophagy. The change in the expression of genes responsible for organelle biogenesis and mitophagy also correlates with the level of mtDNA, which makes it possible to assess the mitochondrial mass in the tissue (Figure 7F). One can see that the level of mtDNA in the skeletal muscles of *mdx* mice was significantly reduced and that NS1619 did not affect the levels. At the same time, in the skeletal muscles of wild-type mice treated with NS1619, we observed a decrease in the level of mtDNA compared with the control animals, which correlated with a decrease in the expression of *Ppargc1a* and mitophagy genes under the action of this agent.

### 3.5. NS1619 Attenuates Skeletal Muscle Destruction in mdx Mice

Based on the obtained results, one could assume that NS1619 improves some parameters of *mdx* mouse mitochondria, the ultrastructure of organelles, and sarcomere size in skeletal muscles. We assessed whether such an action of this agent could mitigate the progression of pathology in *mdx* mice and influence the intensity of degeneration/regeneration cycles, fibrosis, the leakage of muscle enzymes, and lastly the muscle strength of animals.

**Figure 8:**
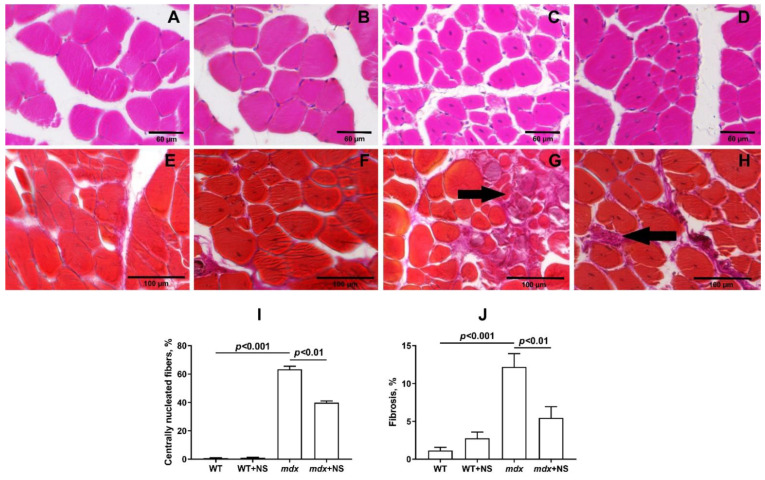
Representative histology images of skeletal muscle tissue (quadriceps) showing myofibers containing centrally located nuclei (**A**–**D**) and fibrotic area ((**E**–**H**), black arrows) from the WT (**A**,**E**), WT + NS1619 (**B**,**F**), *mdx* (**C**,**G**) and *mdx* + NS1619 (**D**,**H**) groups of mice. The bars are equal to 60 (**A**–**D**) and 100 μm (**E**–**H**). Diagram (**I**,**J**) show the percentage of myofibers containing centrally located nuclei and the amount of interstitial fibrosis in the skeletal muscles of the experimental animals. The data are presented as m ± SEM. The level of fibrosis was evaluated as the percentage ratio of the hematoxylin van Gieson (HvG) staining areas (pink) that represented collagen and other connective tissue elements in the common area of the tissue, wherein at least 10 sections were analyzed for each sample. Quantitative data of the centrally nucleated fibers from H&E staining are from an average of 8–10 unique visual fields from each animal and expressed as a percentage of the total number of myofibers counted.

To examine the intensity of degeneration/regeneration cycles and the dystrophic histopathology of the muscles from untreated and NS1619-treated *mdx* mice, we scored myofibers with central nuclei (CNF) in H&E-stained sections in all groups. The histological analysis of the *mdx* mice muscles showed the extensive centrally nucleated fibers, a hallmark feature of the ongoing degeneration and regeneration of muscles [45] (Figure 8A–D,I). Treatment with NS1619 resulted in a significant decrease in this parameter.

The level of fibrosis development in the skeletal muscles of all experimental and control groups was compared. The highest level of fibrosis was observed in the *mdx* group of animals as well as in the *mdx* + NS group. For the same groups, the largest values of variance were found, which characterizes the increased reaction norm in these individuals. Statistically significant differences were found between groups of mice with Duchenne muscular dystrophy and all other groups. Thus, NS1619 reduces the level of fibrosis in the skeletal muscles of *mdx* mice, which also indicates the relief of muscle pathology (Figure 8E–H,J).

**Figure 9:**
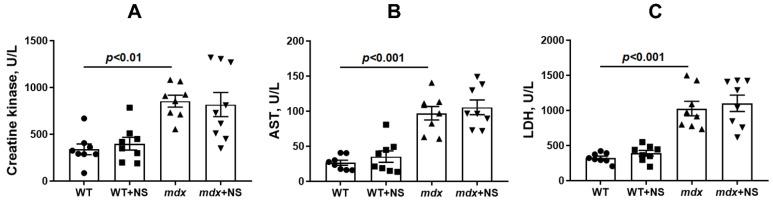
The effect of NS1619 on the activity of creatine kinase (**A**), AST (**B**), and LDH (**C**) in the blood serum of mice. The data are presented as m ± SEM.

One of the main diagnostic criteria indicating the development of DMD is a high level of muscle enzymes in the blood serum, such as creatine kinase, lactate dehydrogenase (LDH), and aspartate aminotransferase (AST). As shown earlier [23,29] and confirmed in this work, dystrophin-deficient animals are characterized by a significant increase in the activity of these markers compared with wild-type mice (Figure 9), which indicates damage to the membranes of the *mdx* muscle fibers. Interestingly, despite the beneficial effect, the use of NS1619 in general did not affect the level of these enzymes in the blood serum of mice.

**Figure 10:**
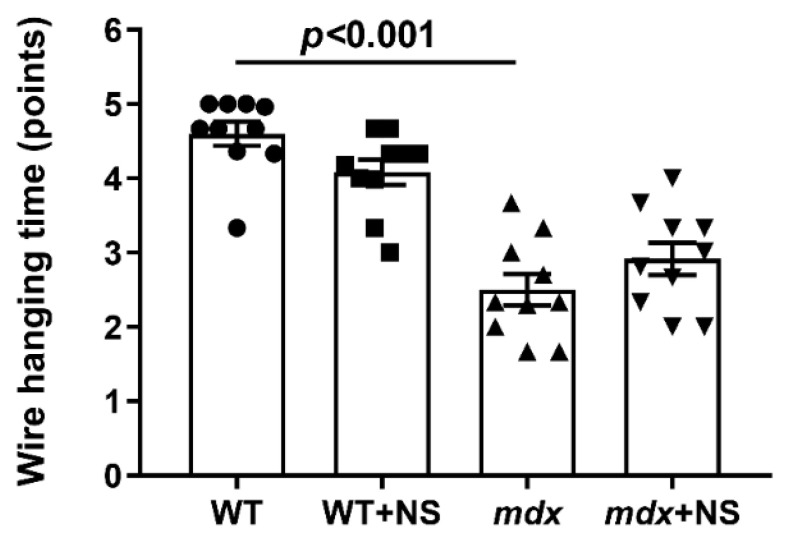
The effect of NS1619 on the results of the wire-hanging test, reflecting the muscle function and endurance of the studied groups of mice. The data are presented as m ± SEM.

In addition, we evaluated the muscle function of the experimental groups of mice in accordance with the generally accepted protocol (the wire-hanging test) [39]. It is known that dystrophin-deficient animals show a low ability to stay on the horizontal bar [17,18,23,29] (Figure 10). This indicates a decrease in muscle strength and endurance of dystrophin-deficient animals compared with WT mice. In this case, NS1619 administration had no effect on the animals’ endurance.

## 4. Discussion

Dystrophin and the dystrophin-associated glycoprotein complex as a whole are known to play an important role as molecular scaffolds for coordinating the assembly of various signaling molecules, including ion channels that ensure the normal functioning of skeletal muscles [3,4]. Therefore, it is not surprising that the loss of these structures observed in both Duchenne muscular dystrophy patients and model animals, along with muscle fiber malfunction, leads to a dysregulation of ion homeostasis. These events are facilitated by changes in the level and function of a number of membrane ion channels, including voltage-gated Na^+^ and Ca^2+^ channels, inwardly rectifying K^+^ channels, and TRP cation-permeable channels [4]. Moreover, it can be assumed that this also leads to a change in the activity of ion channels localized in intracellular organelles and, in particular, in the sarcoplasmic reticulum and mitochondria, the main calcium depots of skeletal muscles [46,47]. These organelles show significant rearrangements in the calcium-transporting protein complexes fixed at the gene level. All these changes contribute to organelle dysfunction, decreased respiratory efficiency and ATP production, dysregulation of intracellular Ca^2+^ buffering, loss of homeostasis, and rapidly progressive Ca^2+^-induced degeneration of skeletal muscle [15,16,17].

Along with the dysregulation of calcium homeostasis, we also noted a significant decrease in the efficiency of potassium ion transport and the total level of this ion in the skeletal muscle mitochondria of dystrophin-deficient animals (Figure 1). Potassium transport through the inner mitochondrial membranes is known to play a central role in triggering cytoprotection and is mediated by a number of membrane transporters whose biophysical properties as well as pathophysiological roles have been described in excellent reviews [30,31]. In this work, we focused on the calcium-activated potassium channel (BK_Ca_) and particularly on mitoBK_Ca_ localized in the inner mitochondrial membrane. BK_Ca_ localization in mitochondria is a result of the VEDEC splice variant from *Kcnma1* gene [41]. We found that VEDEC expression was significantly reduced in the skeletal muscles of *mdx* mice compared with wild-type animals (Figure 2), which may indicate a decrease in the level of mitoBK_Ca_ in dystrophin-deficient animals. Taking into account the known role of mitoBK_Ca_ in protecting mitochondria from ROS overproduction and maintaining the calcium-buffering function of organelles [31], one could assume that this may also contribute to complex organelle dysfunction, which is manifested in the suppression of oxidative phosphorylation (Table 2), a decrease in resistance to MPT pore opening (Figure 3), the development of oxidative stress (Figure 4) and the disturbance of the ultrastructure of organelles (Figure 5). 

It is well-known that the modulation of mitoBK_Ca_ has a cytoprotective effect on cardiac ischemia and reperfusion (I/R) injuries [48,49,50] and shows a neuroprotective effect [51]. Given the similar biophysical and pharmacological properties of BK_Ca_ [31], we should expect similar effects on other pathologies, which in particular affects skeletal muscles. In this work, we evaluated the effects of the NS1619 benzimidazole derivative BK_Ca_ activator, which has a cytoprotective effect [31] on mitochondrial dysfunction and skeletal muscle status in dystrophin-deficient mice. It is well-known that already a single intraperitoneal administration of 1 mg/kg of NS1619 has a protective effect on the heart muscle in a mouse I/R model [49,50]. Considering the hereditary nature of Duchenne dystrophy and the corresponding severity of this pathology, in this pilot study we chose a dose of 0.5 mg/kg body weight and chronic administration of the drug for 4 weeks in 8-week-old dystrophin-deficient mice, showing a stable slow development of skeletal muscle pathology. We found that this dose of NS1619 significantly improves the rate of potassium ion transport and increases the total amount of this ion in the skeletal muscle mitochondria of *mdx* mice (Figure 1). This action of NS1619 is also accompanied by the normalization of the mitoBK_Ca_ mRNA level in the skeletal muscle (Figure 2). Thus, NS1619 is indeed able to normalize the homeostasis of potassium ions in the mitochondria of skeletal muscles in dystrophin-deficient mice, and this effect may be due to the activation of mitoBK_Ca_ and an increase in its level in the inner membrane of the organelles.

Improving potassium transport across the inner mitochondrial membrane, including by mitoBK_Ca_ activation, may reduce the intensity of ROS production in and increase the resistance of organelles to the induction of a calcium-dependent MPT pore [31]. In this work, we found that the long-term administration of NS1619 to dystrophin-deficient mice led to a significant improvement in the calcium retention capacity of skeletal muscle mitochondria, reflecting resistance to the opening of the MPT pore (Figure 3). Additionally, we observed a decrease in the level of lipid peroxidation products (Figure 4), which may also indicate a decrease in the intensity of oxidative stress in the skeletal muscles of *mdx* mice.

It is noted that the knockout of mitoBK_Ca_ leads to disruption of the ultrastructure of mitochondria, namely to their swelling and loss of continuity of IMM compared with wild-type cells expressing mitoBK_Ca_ [52]. Moreover, the loss of channel properties of mitoBK_Ca_ is accompanied by the loss of mitochondrial content and disruption of mitochondrial dynamics [51,53]. All these phenomena were also observed in the case of dystrophin-deficient animals. In particular, we noted swelling of the organelles (Figure 5 and Figure 6) and a decrease in the level of mtDNA (Figure 7F). In addition, we noted a decrease in the expression of the *Ppargc1a* and *Mfn2* genes in the skeletal muscles of *mdx* mice, which may indirectly indicate the inhibition of organelle biogenesis, as well as mitochondrial fusion (Figure 7A,C). In this case, the administration of NS1619 and the improvement of the transport of potassium ions in mitochondria also led to the restoration of the ultrastructure of the mitochondria of the skeletal muscles of *mdx* mice and normalization of the size of organelles (Figure 5 and Figure 6C), but did not affect the expression of genes encoding proteins responsible for mitochondrial dynamics, biogenesis, mitophagy, and mtDNA level (Figure 7). In this case, we also note the normalization of muscle sarcomere size altered in *mdx* mice (Figure 6A,B).

Particular attention should be paid to the effect of NS1619 on the functioning of mitochondria in the skeletal muscles of wild-type mice, which normally express dystrophin and as a result demonstrate a normal level of mitoBK_Ca_ expression. One can see that treatment with NS1619 leads to a significant suppression of respiration of wild-type mice skeletal muscle mitochondria in almost all functional states, which is also accompanied by a 1.4-fold decrease in the respiratory control ratio (Table 2). In the case of *mdx* mice, we also noted this trend; however, against the background of reduced respiratory activity of mitochondria in these animals (compared to WT), the inhibitory effect of NS1619 was less pronounced. Moreover, due to a decrease in the respiration rate in state 4 (characterized as a state without any ATP usage/production), the effect was accompanied by an increase in the respiratory control ratio. NS1619 is known as an agent with off-target activity, and a number of studies have indicated the ability of this agent to suppress the functioning of mitochondrial respiratory chain complexes [54,55]. One could assume that this effect of NS1619 not only contributes to the suppression of mitochondrial respiration in the skeletal muscles of wild-type mice (Table 2), but also contributes to a decrease in the calcium retention capacity (Figure 3). In addition, in this study we noted a significant change in the expression of genes that reflect the level of biogenesis, mitophagy, and the state of mitochondrial dynamics. Indeed, the skeletal muscles of WT mice treated with NS1619 show a significant increase in the expression of the *Drp1* gene responsible for mitochondrial fission (Figure 7B), a reduction in the expression of *Ppargc1a* responsible for organelle biogenesis (Figure 7C), and a significant decrease in the mtDNA level (Figure 7F). It should be noted that NS1619 also upregulated the expression of the *Drp1, Pink1*, and *Parkin* genes (Figure 7D,E) responsible for mitophagy. The latter may be an adaptive response that promotes the selective removal of poor-quality skeletal muscle mitochondria that exhibit swelling (Figure 5B and Figure 6C) and whose function was possibly downregulated by NS1619. In this case, a slight decrease in the size of muscle sarcomeres can also be noted (Figure 6A,B).

It has been previously shown that improving mitochondrial function by inhibiting MPT pore opening or normalizing the organelle biogenesis alleviates the destructive processes in the skeletal muscle of dystrophin-deficient animals and corrects muscle function [23,25]. In the present work, we also noted an improvement in muscle pathology under the action of the mitoBK_Ca_ activator NS1619, which manifested itself in a decrease in the intensity of degeneration/regeneration cycles in muscle fibers, as evidenced by a decrease in the level of centrally nucleated fibers, as well as in the level of fibrosis in the skeletal muscles of NS1619-treated *mdx* animals. On the other hand, it did not affect the leakage of muscle enzymes (CK, AST and LDH) into the blood serum of *mdx* mice, which indicates damage to muscle fiber membranes and promotes general inflammation. This intriguing contradiction may be due to a different expression of mitoBK_Ca_ in different types of skeletal muscles and the heart [31], which may accordingly indicate some muscle-specific action of NS1619 that does not lead to a general decrease in leakage of muscle enzymes, which requires further study. In support of this, we also did not note the effect of such therapy on the loss of muscle strength in dystrophin-deficient animals despite the muscle sarcomere size normalizing. These data also confirm the important role of maintaining the integrity of muscle fiber membranes and treating inflammation to improve muscle function in Duchenne dystrophy cases [12]. Indeed, the treatment of inflammation with corticosteroids is the main approach in the current therapy of DMD [56]. Thus, NS1619 certainly has a therapeutic effect in the treatment of Duchenne dystrophy, at least in dystrophin-deficient mice. However, the effect of this treatment is incomplete and requires the search for the optimal concentration and mode of administration, as well as the use of additional approaches that reduce the intensity of inflammation.

## 5. Conclusions

The results of this work again demonstrate the dysregulation of ion homeostasis in the skeletal muscle mitochondria of *mdx* mice, which was initially caused by the loss of the dystrophin protein and, accordingly, the dystrophin-associated glycoprotein complex. Along with well-known dysfunctional rearrangements in the calcium-transporting and accumulating systems of mitochondria, we noted a significant decrease in mitoBK_Ca_ expression, which can contribute to the reduction of potassium ion transport in skeletal muscle mitochondria and the total level of this ion. This in turn can make a significant contribution to the complex dysfunction of skeletal muscle mitochondria in dystrophin-deficient animals and disrupt their ultrastructure. The administration of the benzimidazole derivative BK_Ca_ activator NS1619 normalized mitoBK_Ca_ expression levels, as well as the potassium transport rate and ion content in organelles; it also contributed to the improvement of calcium retention capacity, a decrease in the intensity of oxidative stress in the skeletal muscle mitochondria of *mdx* mice, and the normalization of the ultrastructure of organelles and muscle sarcomere size. These effects of NS1619 led to a decrease in the intensity of degeneration/regeneration cycles and level of fibrosis in the skeletal muscles of *mdx* mice, but had no effect on the leakage of muscle enzymes or on muscle strength loss. Thus, the mitochondrial approach associated with the improvement of potassium ion homeostasis can be used as a secondary therapy for the treatment of Duchenne muscular dystrophy, but only in combination with other methods that are primarily aimed at normalizing calcium homeostasis, organelle biogenesis, and reducing generalized inflammation.

Undoubtedly, this study contains some limitations. In particular, samples from one mouse muscle were quite small and were required for many experiments. Further studies should be conducted to broaden the findings of this pilot study. This implies an additional validation of the level of proteins by the Western blotting. We also note the need to evaluate the dose–response curve, especially considering the identified side effects of NS1619, which has low selectivity for mitoBK_Ca_ and demonstrates a pleiotropic effect [31], and this also requires the search for new adequate modulators of this channel.

## Figures and Tables

**Table 1:** Oligonucleotide sequences of the RT-PCR primers.

Gene	Forward (5′→3′)	Reverse (5′→3′)
*BKCa-DEC*	GGTTTACAGATGAGCCGGATA	CATCTTCAACTTCTCTGATTGG
*D* *rp1*	TTACAGCACACAGGAATTGT	TTGTCACGGGCAACCTTTTA
*Mfn2*	CACGCTGATGCAGACGGAGAA	ATCCCAGCGGTTGTTCAGG
*Pink1*	TTGCCCCACACCCTAACATC	GCAGGGTACAGGGGTAGTTCT
*Parkin*	AGCCAGAGGTCCAGCAGTTA	GAGGGTTGCTTGTTTGCAGG
*Ppargc1a*	CTGCCATTGTTAAGACCGAG	GTGTGAGGAGGGTCATCGTT
*Rplp2*	CGGCTCAACAAGGTCATCAGTGA	AGCAGAAACAGCCACAGCCCCAC
*Nd4*	ATTATTATTACCCGATGAGGGAACC	ATTAAGATGAGGGCAATTAGCAGT
*Gapdh*	GTGAGGGAGATGCYCAGTGT	CTGGCATTGCTCTCAATGAC

**Table 2:** The effect of NS1619 on the parameters of respiration and OXPHOS of the skeletal muscle mitochondria of the studied groups of mice.

Group	V Respiration, nmol O_2_ * min^−1^ * mg^−1^ Protein				RCR	*ADP/O*
	State 2	State 3	State 4	State 3U_DNP_		
WT	23.3 ± 1.7	199.9 ± 7.0	44.2 ± 3.3	217.7 ± 8.4	4.7 ± 0.2	1.2 ± 0.1
WT + NS	18.4 ± 1.2 *	116.4 ± 8.6 *	34.1 ± 0.9	141.7 ± 8.0 *	3.4 ± 0.3 *	1.2 ± 0.1
*mdx*	20.0 ± 1.3	168.2 ± 7.6 *	55.5 ± 7.9	189.4 ± 6.8 *	3.8 ± 0.5	1.2 ± 0.1
*mdx* + NS	21.4 ± 0.8	147.0 ± 5.4 *	34.1 ± 1.9 #	171.6 ± 5.0 *	4.5 ± 0.3	1.4 ± 0.1

Respiration of mitochondria in state 3 was initiated by 200 μM of ADP. Mitochondrial respiration in the 3U_DNP_ state was induced by 50 µM of DNP. The data are presented as m ± SEM. * *p* < 0.05 versus WT group. # *p* < 0.05 versus *mdx* group.

## Data Availability

The data presented in this study are available upon request from the corresponding author.
